# Acceptance of e-learning in higher education: The role of task-technology fit with the information systems success model

**DOI:** 10.1016/j.heliyon.2023.e13751

**Published:** 2023-02-18

**Authors:** Ibrahim Youssef Alyoussef

**Affiliations:** Faculty of Education, Education Technology Department, King Faisal University, Al Ahsa, 31982, Saudi Arabia

**Keywords:** E-learning, Perceived enjoyment, Information quality, E-learning benefit

## Abstract

The COVID-19 global epidemic has compelled higher education institutions to reconsider their teaching methods. Because of this public health emergency, universities in higher education have adopted e-learning techniques as a solution to face-to-face education. Thus, e-learning has emerged as a critical technology in education at higher education institutions. Nonetheless, the effectiveness of e-learning systems is largely dependent on students' adoption of such systems. The study aims to evaluate the usefulness of task-technology fit (TTF) with the information system success model (ISSM) in perceiving students' adoption of e-learning with the goal of encouraging them to adopt e-learning in the context of higher education. The study employed a quantitative approach, and a theoretical model was evaluated with proposed hypotheses to find the relationships between the constructs. A questionnaire based on TTF and ISSM was distributed among the students, and 260 valid responses were received using a sample random sampling technique. Data was analyzed with the help of SPSS and Partial Least Squares-Structural Equation Modeling (PLS-SEM). After analyzing the data, it was found that perceived ease of use, perceived usefulness, system use, and task technology fit of e-learning are positively and significantly influenced by system quality, information quality, perceived enjoyment, technology characteristics, and task characteristics. The results of TTF and ISSM on system use show a positive effect on e-learning benefits in educational institutions, with all male and female students completely satisfied with the use of e-learning systems. As a result, we advise students to use e-learning systems for educational purposes and should have motivated them to do so through lecturers at higher-level educational institutions.

## 1 Introduction

The Since the need for e-learning has been rising year over year, the Internet and technology’s rapid development have encouraged the education industry to embrace Internet-based learning resources from primary to higher education. E-learning in this context refers to a concept in education that makes use of digital technology and gadgets to distribute learning materials and promote distance learning [1,2]. Due to its improved capacity for providing high-quality teaching, the education sector is among those promising and lucrative industries that are most affected by the adoption of technology. The level of e-learning advantage, however, affects the e-learning environment. Universities and colleges must promote a collaborative learning environment if they want to increase student performance and support their higher levels of information acquisition [3]. Education platforms have undergone major change as a result of the growth of e-learning mechanisms in higher education institutions, with the emphasis now mostly on the students rather than the lecturers [4]. Numerous universities have implemented e-learning as an innovative method of teaching [5]. Meanwhile, learners have become more diverse, and there is a growing demand for e-learning programs to help them with their learning [6,7]. Integrating information technology into teaching and learning [8,9] allows for classroom restrictions to be reduced and students to have more opportunities to communicate with each other, resulting in more effective learning [10,11].

According to Costley and Lange [12], e-learning can have a greater impact on students' academic performance, achievements, and levels of satisfaction than traditional classroom instruction. E-learning has gained greater attention in the academic system in the last few years because of its useful, flexible, and cost-effective characteristics [13], and it has significantly changed conventional learning and teaching methods in many developed and developing nations. Previous research has found that advanced technologies positively affect the adoption of e-learning [14]. According to Ref. [15], the acceptance of technology can predict access to knowledge and information and develop an individual’s belief in the development of technology. Rejecting technology can raise worries that can stifle decision-making in general. This has become essential for both students and lecturers to have access to all of the technological tools required for effective learning and teaching practices [16,17].

These tools are designed to help students feel more confident in their ability to gain knowledge on their own. Research has been done to investigate the effects of personal factors on the use of ICT in e-learning. Some of them reveal that implementing e-learning usually requires improvements in performance and greater confidence on the part of both students and instructors [[18], [19], [20]]. Adoption and implementation of new technologies required to develop e-learning in HEIs take time, require training, and require subjective norms and an institutional willingness to accept new technologies. Surprisingly, [21,22] discovered that innovative technologies alone cannot produce the desired academic changes; ongoing human investment in training is also required. According to Ref. [23], students struggle to shift to the online platform because of a shortage of internet service and other resources to enable e-learning. When users have fewer resources or less ability to access e-learning, they have an unexpected experience on the online platform [24,25].

However, the COVID-19 epidemic may have increased the adoption of e-learning systems more than any other circumstance, mostly in developing countries. The skills of students and lecturers to use various e-learning systems are important for the implementation of e-learning. The effectiveness of e-learning requires the collaboration of students, who must shift from conventional course offerings and manage the most recent technological terrain, which has unique requirements [26,27]. Costly data charges and other social inequality worsen the gap in reaching a quality education, and these concerns are broadening the technology gap [24,28,29]. In their study, Nigerian students reported that the higher cost of ICT devices had a negative impact on e-learning acceptance. According to Ref. [30], one of the most important predictors of online course adoption is instructor feedback. In addition, [31] discovered in their study that most students prefer blended learning, with less than 5% preferring conventional face-to-face learning.

Similarly, a study conducted in the context found that costly internet data restrains the learners' access to entirely taking part in e-learning [32,33]. Furthermore, [34] claimed that students from developing countries with limited facilities found it difficult to fully integrate into the digital environment. Initially, academics tried to resist the shift from face-to-face to e-learning because they believed that in-class students were better than online students. Previous research has used a variety of theories, including DeLone and McLean’s model, the TAM, IDT, and UTAUT, to examine the behavioral patterns of e-learners. Several of them thought about the reasons for and barriers to the adoption of e-learning [[35], [36], [37]]. Therefore, in order to investigate how e-learning assists individuals in higher education, we tried to present an extended TTF model as well as DeLone and McLean’s approach. Since system use is a crucial component of system success, Ref. [38] argue that it is crucial to look into the linkages between e-learners' experiences, intentions, and behavioral intentions for system use. The TTF approach, first proposed by Goodhue and Thompson [39], focuses on matching technology to a task [40].

According to the TTF, the adoption of technology is determined by how well a novel technology appears to fit the needs of specific tasks [39]. It also broadens the ISSM by taking into account how the tasks influence usage intention [41]. Moreover, there is a scarcity of research on university students' intentions to adopt e-learning during the global epidemic that uses the TTF and ISSM models with perceived enjoyment as an external factor of the model, especially in developing countries such as education, where e-learning was not widely used at universities prior to the pandemic. To address these gaps, the study attempts to validate an integrated TTF with ISSM as a means of assessing the influential factors influencing undergraduate students' willingness to use e-learning for teaching and learning.

Hence, this study wants to know two things: (1) what factors influence undergraduate students' decisions to use e-learning for learning? (2) What are the interrelationships between these factors? The study aims to inspect the elements that influence undergraduate students' willingness to use e-learning for learning, recognize the relationships between these factors, and empirically test an integrated TTF with ISSM. This research contributes to a better understanding of undergraduate students' intentions to adopt e-learning by integrating variables such as PEOU, PU, task-technology fit, and system use, which can be used as a theoretical model in future studies in the area of education. Besides that, this research offers universities and lecturers a set of recommendations for motivating students to use e-learning for learning and achieving better results.

### 1.1 E-learning during Covid-19

E-education, distant learning, and e-learning are other terms for e-learning. “A wide range of applications and procedures that utilize accessible electronic media and resources to give occupational education and training” is how the authors [42] define e-learning. Researchers define e-learning as “the use of various technology tools that are web-based, web-distributed, or online competent for education” [43,44]. It is evident that during the pandemic COVID-19 age, all students and instructors are confused about how to carry out the learning process [45,46]. Due to an unexpected scenario in which the entire campus was briefly shuttered [47]. Therefore, professors and students were obliged to study from home. As a result, a solution to connect students and lecturers in order to continue the learning process even if they are unable to meet on campus is required. The COVID-19 pandemic period has ushered in rapid development. As a result, the planning for dealing with diverse changes is not optimal. This is something that the educational community is also aware of [48].

The government has established a low bar for readiness to study online (e-learning). In this example, the professor is expected to aim to produce learning that will continue even when they are not on campus. Get acquainted with online learning. In this research, a study is presented that aims to determine whether students are ready to study online and what challenges they face when coping with e-learning in the wake of the COVID-19 outbreak [49,50]. The study’s conclusions state that learners must have access to a mixed strategy that blends conventional and online instruction. Another study is described, with the aim of improving learners' capacity for autonomous learning by exploring the e-learning process among those who are conversant with web-based technology [51]. According to the survey’s results, e-learning has become more and more popular among students across all academic institutions during the COVID-19 pandemic lockdown. Changes in the learning environment, however, appear to be more than a matter of choice in the current COVID-19 pandemic condition, when people must keep their distance from one another. As a result, many academics and educators are currently analyzing the data surrounding remote e-learning and identifying the best method to implement such an approach to continue students' education and training in this tumultuous social environment [[52], [53], [54]].

**Fig. 1:**
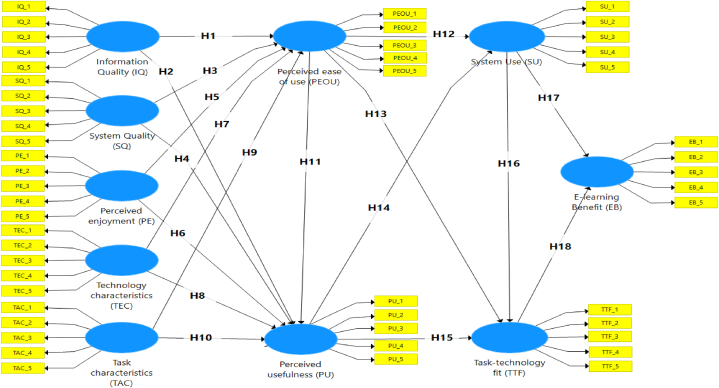
Research model.

The major goal of this work is to propose and test a model to integrate task-technology fit and ISSM elements that affect how e-learning systems are used as well as the task-technology fit. The review of the literature focused on ten constructs based on the study model: Combining the TTF with the success model of information systems, a research model was developed with variables that are information quality, system quality, technology characteristics, task characteristics, and perceived enjoyment factors. It is asserted that the e-learning benefit of adopting e-learning can be affected by a series of significant factors, including information quality, system quality, technology characteristics, task characteristics, and perceived enjoyment. Additionally, it is argued that information quality has an effect on both PEOU and PU, task-technology fit, system use, system quality, technology characteristics, task characteristics, and perceived enjoyment. Consequently, in the present study, seven factors influencing TTF and ISSM acceptance of e-learning benefits were examined, such as information quality, system quality, technology characteristics, task characteristics, perceived enjoyment, perceived usefulness, perceived ease of use, system use, and task-technology fit (see Fig. 1).

The e-learning platform is the principal application of IT in education. There are currently no available standards for e-learning system views [55]. The use of online learning and education tools to reduce learning loss Web-based technology’s application was started by Electron Learning [56]. Nowadays, e-learning is employed all over the world [[57], [58], [59]] defined e-learning as the delivery of knowledge through technology and the internet. The majority of universities and colleges have a well-established e-learning platform for their students [60]. The potential of e-learning to produce more notable effects on all study modes, including full-time, part-time, and distance learning, is its most notable impact on students at higher educational institutions. According to Ref. [56], most graduate students take classes part-time since some of them have jobs. As a result of their time constraints, e-learning significantly contributes. Massive open online courses (MOOCs) have been advancing e-learning since 2012 [61,62]. MOOCs are classes that were made available in higher education to assist people in developing their professional abilities [63]. Recently, a few of these universities also decided [56] to activate the e-learning portals [64]. The work carried out by figured out problems related to the Learning Management System (LMS) in King Faisal University universities, for instance the limited availability of trained experts, inadequate WIFI coverage and connection speed, disadvantages of infrastructure and facilities, poor quality of contents, disability to use the system, and lack of knowledge among students. In this research, the success of e-learning adoption among students will be compared. The importance of e-learning from the perspective of King Faisal University students will also be explored.

## 2 Theoretical model and hypotheses

### 2.1 Task-technology-fit model

The task-technology fit (TTF) concept contends that performance and acceptance are influenced by how well a task and the technology being used to complete it “fit” together [39]. Learning management systems (LMS) and digital video tools are two examples of the technology in the education field to which this approach has been applied [65,66]. These studies indicate that performance improves when technology is ideally adapted to the users' abilities and tasks. The task-technology fit (TTF) model is a popular theoretical framework for analyzing the effects of information technology on performance, gauging usage effects, and determining the compatibility of task and technology features. The task-technology fit, which in turn affects users' performance and utilization, can be influenced by both task features and technological qualities. TTF has been extensively investigated and used in a variety of information systems since it was first proposed [67]. TTF has been the subject of research in a number of contexts, but e-learning has received relatively less attention. Up to this point, it hasn’t been determined if or how well a solid task-technology fit will affect a user’s adoption of e-learning. The TTF model does not take into account social dynamics in the context of e-learning, which would limit its potential to forecast the use of social networking technology. By increasing the constraint with social motivation and gaining knowledge from social influence and acknowledgment, the problem can be solved.

### 2.2 Information system success model (ISSM)

Delone and McLean [68] suggested the ISSM as a way to gauge the success of IS in organizations and determine the net benefit. They argued that a multidimensional, symbiotic paradigm underlies IS success. Therefore, understanding and managing the interactions between those dimensions is essential. Numerous academics then proposed some changes to this paradigm [69]. As a result, [70] revised their old model with the ISSM in 2003, as shown in Fig. 1. They also incorporated some of the adjustments that scholars had suggested. They made the decision to increase the importance of the service quality, customer satisfaction, usage intent, and net benefit dimensions. According to the new model, the key success elements for implementing ISSM to achieve net benefits are service, system, information quality, system utilization, and user happiness. The researchers suggested that service, system, information, and quality are the determining factors of ISSM’s continued use if a successful evaluation of the system is sought. User satisfaction is a result of the positive or negative benefits that will determine how much IS is used [70].

#### 2.2.1 Information quality (IQ)

Information quality is the level of output produced by an information system, including its relevance, timeliness, scope, and accuracy [71]. Information quality is a vital and crucial aspect in determining the efficacy of information and e-learning systems due to the crucial role information plays in accomplishing educational goals and the enormous obstacles brought on by low IQ [16]. In order to ascertain the relationship between information quality and user experience, the Delone and McLean [71] model was applied. The success dimension’s representation of the output quality characteristics of an IS is content and information quality [72,73], with e-learning as an illustration. As a result, it includes indicators that concentrate on the quality of the information that the system generates and its applicability to consumers. A common belief is that the quality of the information is a major factor in determining how satisfied users are and whether they plan to employ e-learning techniques [35,[74], [75], [76], [77]]. As a result, it is assumed in this study that content and information quality have a beneficial impact on reported PEOU and PU.

Hypothesis 1 (H1): Information quality is positively and strongly related to perceived ease of use.

Hypothesis 2 (H2): Information quality is positively and strongly related to perceived usefulness.

#### 2.2.2 System quality (SQ)

System quality has been defined as the information system’s usability, accessibility, utility, complexity, and response time [71]. Davis' TAM and DeLone and McLean’s [71,78] information systems success model both use actual system utilization as a metric (1989). The variable “usage” has a significantly negative impact on the advantages of the system, according to Ref. [73] systematic review of the literature. Prior studies found a significant connection between system utilization and advantages [35,79]. It has been demonstrated that deploying e-learning technologies to deliver employee training sessions will directly and favorably affect the company’s net benefits [35]. Numerous other research findings revealed findings that were similar to these [80]. In light of this, we anticipate that adopting the system would increase students' access to better knowledge, time savings, and effective management of the learning processes. The following theory is put forth and is backed up by earlier research:

Hypothesis 3 (H3): SQ is positively and strongly related to PEOU.

Hypothesis 4 (H4): SQ is positively and strongly related to PU.

#### 2.2.3 Perceived enjoyment (PE)

The concept of enjoyment is consistent with inherent inspiration [81], and it is defined in the context of information system utilization as the degree to which the act of using a specific technology is seen as enjoyable in and of itself, regardless of any outcomes of system use [82,83]. Perceived enjoyment is essential in assessing the adoption of e-learning. Earlier studies, according to Abdullah and Ward [84,85], found that PE had a substantial impact on the PEOU and PU for e-learning. Numerous different studies have found that PE improves students' intentions to use e-learning [35,79,83]. Moreover, Abdullah and Ward [85] reported a substantial and optimistic relationship between PE and PEOU for e-learning in eight out of eleven research studies. The same is true between PE and PU, as is evident from eight out of eight studies (100%). It is thus declared that if an individual enjoys an e-learning system, he or she will have a positive attitude toward the system’s ease of use and usefulness, as well as a stronger intention towards the use of the system [86].

Hypothesis 5 (H5): PE is positively and significantly related to PEOU

Hypothesis 6 (H6): PE is related to PU in a positive and significant way.

#### 2.2.4 Technology characteristics (TEC)

The system that students currently utilize to complete their assignments possesses certain technological qualities [39]. TTF is the extent to which a technology supports users in conducting their group of tasks; an individual converting inputs into outputs generally defines tasks as the actions performed; and technology is regarded as a medium in performing the users' tasks [39]. Furthermore, performance impact relates to an individual’s achievement of a portfolio of duties, and higher performance recommends a combination of increased efficacy, success, and/or quality [39]. TTF is explained as the extent to which the system aids the student in completing his or her portfolio of learning activities in accordance with Goodhue and Thompson’s [39] definition and [87] definition. Earlier studies have generally demonstrated the impact of task characteristics and technology characteristics on TTF in a range of scenarios [39,40,88]. As a result, this study contends that TC and TEC are crucial factors in determining how well students perceive a cloud-based e-learning system’s TTF. As a result, the research suggests the following theories:

Hypothesis 7 (H7): TEC is positively and significantly related to PEOU.

Hypothesis 8 (H8): TEC is positively and significantly related to PU.

#### 2.2.5 Task characteristics (TC)

Users' perceived task needs serve as a defining element of a task, and they were measured using a social structural dimension to determine the task demands [39]. Task characteristics (TC) and their impact on information system utilization have been the subject of numerous studies [39,89,90]. When using the TAM, however, one challenge is that its components may not adequately reflect the different task contexts in which users work. Because of a lack of task emphasis on analyzing IT and its adoption, use, and performance, its application has yielded varied results [91]. Despite the fact that task characteristics are generally included in TAM’s usefulness concept, a more specific addition of this component is required to properly comprehend IT usage [92]. Task equivocality (TE) and task interdependence (TI), according to earlier research on TC and its impact on IS, both affect information technology use and attitude [39]. Task dependency and task ambiguity may both be related to the subjective norm. According to Ref. [91], TI has a significant impact on the subjective norm. In other words, people with higher task interdependence expect greater information technology usage from others.

Hypothesis 9 (H9): A task characteristic is positively and strongly related to PEOU.

Hypothesis 10 (H10): A task characteristic is positively and significantly related to PU.

#### 2.2.6 Perceived Ease of Use (PEOU) and task-technology fit (TTF)

Perceived Ease of Use is described by Davis (Davis, 1989) as “the extent to which the individual believes that employing a certain system would increase his or her job performance.” The degree to which a person believes that using a particular technology would not be difficult to grasp is known as “perceived ease of use” [93]. According to Ref. [94], PEOU is associated with a better elevation of behavioral intention to utilize it, both directly and indirectly. Regarding e-learning, PEOU is a term that refers to a student’s idea that using an e-learning system would be easy. Students' interactions with e-learning are straightforward and understandable [95,96]. Similarly, the usefulness of PEOU will influence students' intentions to adopt the e-learning system, either directly or indirectly. PEOU and BI have a significant positive relationship in this regard. As a result, we developed the following hypothesis:

Hypothesis 11 (H11): PEOU is positively and strongly related to PU.

Hypothesis 12 (H12): PEOU is positively and strongly related to the US.

Hypothesis 13 (H13): PEOU is positively and strongly related to TTF.

#### 2.2.7 Perceived usefulness (PU) and task-technology fit (TTF)

When someone uses new technology, they perceive it as valuable and believe it will help them perform their jobs better [97]. Furthermore, [98] state that PU is an important factor in deciding whether or not to use a particular technology. Students will only accept an e-learning system if they believe that using the specific system will develop their learning skills. From the perspective of e-learning, PU indicates the degree to which students have faith that using an e-learning system would improve their learning efficiency. As a result, PU is supposed to have an effect on the decision to adopt and use the e-learning system, either directly or indirectly (through PEOU). Previous e-learning research found that PU significantly influences users' intention to use the e-learning system [99]. As a result, for this study, the hypothesis is proposed that:

Hypothesis 14 (H14): PU is related to US in a positive and significant way.

Hypothesis 15 (H15): PU is positively and strongly related to TTF.

#### 2.2.8 System use (US) and task-technology fit (TTF)

Actual system usage is a need in both Davis' TAM and the information systems success model developed by Delone and McLean [71]. In their extensive literature review, [73] discovered that “usage” had a moderate relationship with the system’s benefits. Previous research has demonstrated the importance of the link between system utilization and advantages [35,79]. It has been demonstrated that using e-learning to deliver training courses to employees has a direct and considerable impact on the business’s net benefits [35,79]. Other research reports the same results [80]. In light of this, we predict that implementing the system will increase the advantages that students will receive from improved knowledge, time savings, and systematic learning management. The following theory is put forth in this study and is supported by earlier research:

Hypothesis 16 (H16): System use is positively and strongly related to TTF

Hypothesis 17 (H17): system use is positively and strongly related to EB.

#### 2.2.9 Task-technology fit (TTF)

Task characteristics measure how well a technology helps a human do their portfolio of tasks; tasks are widely defined as the activities people take to transform inputs into outputs, while technologies are seen as the means by which people complete their tasks [39]. The necessity for a link between technological attributes and task characteristics forms the basis of the TTF model [39]. Along with TTF, focusing just on students' technological expectations is insufficient for evaluating technology adoption [66]. Students will surely adopt technology if they recognize that it will help them execute their everyday tasks effectively [39]. The paradigm of TTF illuminates the functional features of technology use. Based on the association between performance expectancy and technological characteristics, the TTF model predicts when users will utilize technology, which is essential since it depends just on students' expectations of technology [39].

Hypothesis 18 (H18): Task-technology fit is positively and strongly related to EB.

#### 2.2.10 E-learning benefit (EB)

Information systems (IS) that contribute to the attainment of an individual, a group of individuals, organizations, and industries are referred to as e-learning benefits. Improvements in decision-making, higher productivity, sales growth, cost savings, enhanced profitability, market efficiency, consumer welfare, creating jobs, and economic growth are just a few examples [100,101]. The most essential performance indicators are e-learning benefits, which represent the balance of positive and negative effects on customers, providers, employees, organizations, marketplaces, manufacturers, economies, and also the culture [71,102]. Moreover, while net benefit was the most significant variable, it could not be studied and comprehended without systems, information, and service quality measurements. The net benefit in this study refers to the e-learning benefit because, when measuring e-learning benefits, we could use the same metric for e-learning benefit content.

## 3 Research methodology

### 3.1 Participants

There were about 275 questions given, and the respondents replied to 260 of them, representing a 94.5% response rate. An in-person examination of these exam papers revealed 15 unanswered questions. They had to be excluded as a result. 260 additional surveys were sent to SPSS. A questionnaire was employed as a data collection method in this study using a quantitative approach. 260 students from colleges took part in the survey. According to higher education scientific research policies, any research work must first receive approval before being carried out. Thus, an ethical clearance was obtained for this study (Ref. No. KFU-REC-2022-OCT-ETHICS267), and data was collected from 260 students, both online and manually, who were chosen at random from King Faisal University students. After stating the intended participants and the study’s goal, ethical permission was obtained from the department of scientific research. This research was conducted at the start of the academic year 2020–2021. The participants were given an introduction to the research before completing the questionnaire, and their participation was completely optional. The survey took about 10–15 min to complete. The participants were chosen from different departments and faculties using a convenience sampling technique. After taking into consideration the missing data and questionnaires that were incomplete, 15 questionnaires were omitted. 260 randomly chosen students from King Faisal University who were enrolled in bachelors, masters, and PhD programs, both domestically and internationally, were the source of the data. According to Krejcie and Morgan (1970) and Hair et al. (2010), who claimed the minimal sample size for quantitative research is (N = 200), the sample size of this study (N = 260) is adequate in light of this. In order to test the fictitiously developed model, information was gathered from currently enrolled students at King Faisal University via a structured physical survey. The sample size was calculated using the formula below.

$$ss=\frac{x^{2}\left(p\right)\left(q\right)}{e^{2}}$$

where SS = Sample Size; Z = 1.55 (95% CI); P = Prevalence Level (0.5 used for sample size required); Q = (1 p); E = Error Term (0.05). By inserting values into the formula, the sample size would be:

$$ss=\frac{1.55^{2}\left(0.50\right)\left(0.50\right)}{0.05^{2}}$$

$$ss=\frac{2.4025\left(0.25\right)}{0.0025}$$

$$ss=\frac{0.600625}{0.0025}$$

240.25 ss =

Sample size plays an important role in the estimation and interpretation of SEM results. The questionnaire was distributed physically, and respondents were asked to return it once completed. As a result, 260 questionnaires were considered for further analysis and coded into SPSS.

### 3.2 Instrument

The authors created a questionnaire survey for this study. The first part includes questions intended to collect respondents' demographic information, such as age, gender, specialization, and year of study. Whereas the second section includes measurement items to assess the variables. For variables, see “Information Quality” and “System Quality.” Five items for each variable were retrieved from the studies of [71,103]. Five items to measure perceived enjoyment are adopted from Refs. [85,104]. Items for “Technology characteristics”: five items were taken from Refs. [39,105]. For the remaining variables, each variable consists of five items: task characteristics retrieved from Refs. [39,105], perceived ease of use form [106,107], perceived usefulness form [106,107], system use from Refs. [108,109], and E-learning benefitA Likert scale of five points was used to assess these factors, with 1 signifying “strongly disagree” and 5 indicating “strongly agree."

## 4 Findings

### 4.1 Data analysis and results

The data collected was evaluated through SmartPLS 3.3.3 for evaluating measurement and structural models. The data was processed in two stages, each evaluating the measurement and structural model of the arrangement as suggested by Ref. [110]. In addition, the authors decided to use PLS-SEM for multiple reasons. First, PLS-SEM is generally used when a study’s goal is to improve on an existing theory [111]. Secondly, it allows for simultaneous analysis of both the measurement and the structural model, resulting in more reliable estimations [112]. Hence, PLS-SEM was the appropriate tool for this study.

### 4.2 Descriptive analysis

There were 187 (71.9%) males and 73 (28.1%) females among the 260 participants. The participants' ages range from 18 to 46 years old. In addition, 16 (6.2%) were between the ages of 18 and 20, while 43 (16.5%) were between the ages of 21 and 24. Furthermore, 99 respondents (38.1%) were between the ages of 25 and 29, whereas 51 respondents (19.6%) were between the ages of 30 and 34. In addition, 34 respondents (13.1%) were between the ages of 35 and 40. The remaining 10 respondents (3.8%) were between the ages of 41 and 45, and 7 respondents (2.7%) were over 46 years old. Based on academic degree and demographic variables of specialization, 72 respondents (27.7%) were from management, 93 (35.8%) from engineering, 47 (18.1%) from science and technology, 37 (14.2%) from social science, and 11 (4.2%) from other fields. See Table 1.

**Table 1:** Demographic profile.

Demographic	Discerption	N	%
Gender	Male	187	71.9
	Female	73	28.1
Age	18–20	16	6.2
	21–24	43	16.5
	25–29	99	38.1
	30–34	51	19.6
	35–40	34	13.1
	41–45	10	3.8
	46 and Above	7	2.7
Specialization	Management	72	27.7
	engineering	93	35.8
	Science & Technology	47	18.1
	Social Science	37	14.2
	others	11	4.2

### 4.3 Measurement model assessment

The reliability (Cronbach’s alpha and composite reliability) and reliability of the proposed should be taken into account while creating the measurement model, according to Ref. [110] (including convergent and discriminant validity) [110]. As presented in Table 2 [113,114], both of these characteristics have satisfactory values above the given threshold of 0.7, as permitted by law. Hence, using this as a reference, the reliability of the construction was customized. To control the validity of convergent, the loading factors and average variance extracted (AVE) were examined [110]. All of the loading factors and AVEs are greater than the recommended minimum values of 0.7 and 0.5, which indicates that the measurement model has convergent validity. These results are reported in Table 2. Cross-loadings, the heterotrait-monotrait ratio (HTMT), and the Fornell-Larker criterion were used to establish the discriminants' validity [110]. The outcomes of the Fornell-Larker criteria were shown in Table 4. The findings show that the square roots of all AVEs are greater than their associations with other constructs [115]. Since the loading indicators of each construct are higher than the loadings of its corresponding constructs, the cross-loading criteria are portrayed in Table 3. Table 5 shows the HTMT ratio results. It shows that the construct’s values for each do not surpass the threshold, which is 0.85. This signifies the validity of the discriminants [116]. No significant errors were found with the developed measurement model’s reliability and validity; hence, proceeding with its evaluation is considered reliable. A 5-point Likert scale with Strongly Agree (5), Agree (4), Undecided (3), Disagree (2), and Strongly Disagree (1) was applied for data assessment, including task-technology fit (TTF) and evaluation information system success model (ISSM) components and demographic data. The above discussions have been extracted from the previous related studies available in the literature.

**Table 2:** Constructs, Items, cross-loading, Composite Reliability, Cronbach Alpha, Average variance extracted.

Construct	Items	IL	CA	CR	AVE
Information Quality (IQ)	IQ 1	0.806	0.902	0.927	0.718
	IQ2	0.870			
	IQ 3	0.860			
	IQ 4	0.875			
	IQ 5	0.825			
System Quality (SQ)	SQ1	0.825	0.901	0.927	0.717
	SQ2	0.856			
	SQ3	0.883			
	SQ4	0.843			
	SQ5	0.826			
Perceived enjoyment (PE)	PE1	0.877	0.908	0.932	0.734
	PE2	0.872			
	PE3	0.886			
	PE4	0.877			
	PE5	0.765			
Technology characteristics (TEC)	TEC1	0.851	0.913	0.935	0.741
	TEC2	0.885			
	TEC3	0.850			
	TEC4	0.867			
	TEC5	0.852			
Task characteristics (TAC)	TAC 1	0.875	0.933	0.949	0.788
	TAC 2	0.882			
	TAC 3	0.890			
	TAC 4	0.900			
	TAC 5	0.891			
Perceived ease of use (PEOU)	PEOU1	0.850	0.906	0.930	0.727
	PEOU2	0.858			
	PEOU3	0.860			
	PEOU4	0.876			
	PEOU5	0.819			
Perceived usefulness (PU)	PU1	0.763	0.802	0.863	0.559
	PU2	0.782			
	PU3	0.754			
	PU4	0.730			
	PU5	0.706			
Task-technology fit (TTF)	TTF1	0.768	0.839	0.885	0.606
	TTF 2	0.733			
	TTF 3	0.788			
	TTF 4	0.829			
	TTF5	0.771			
System Use (SU)	SU1	0.847	0.915	0.937	0.748
	SU2	0.892			
	SU3	0.885			
	SU4	0.886			
	SU5	0.812			
eLearning Benefit (EB)	EB1	0.822	0.867	0.904	0.653
	EB2	0.745			
	EB3	0.828			
	EB4	0.825			
	EB5	0.819

**Table 3:**
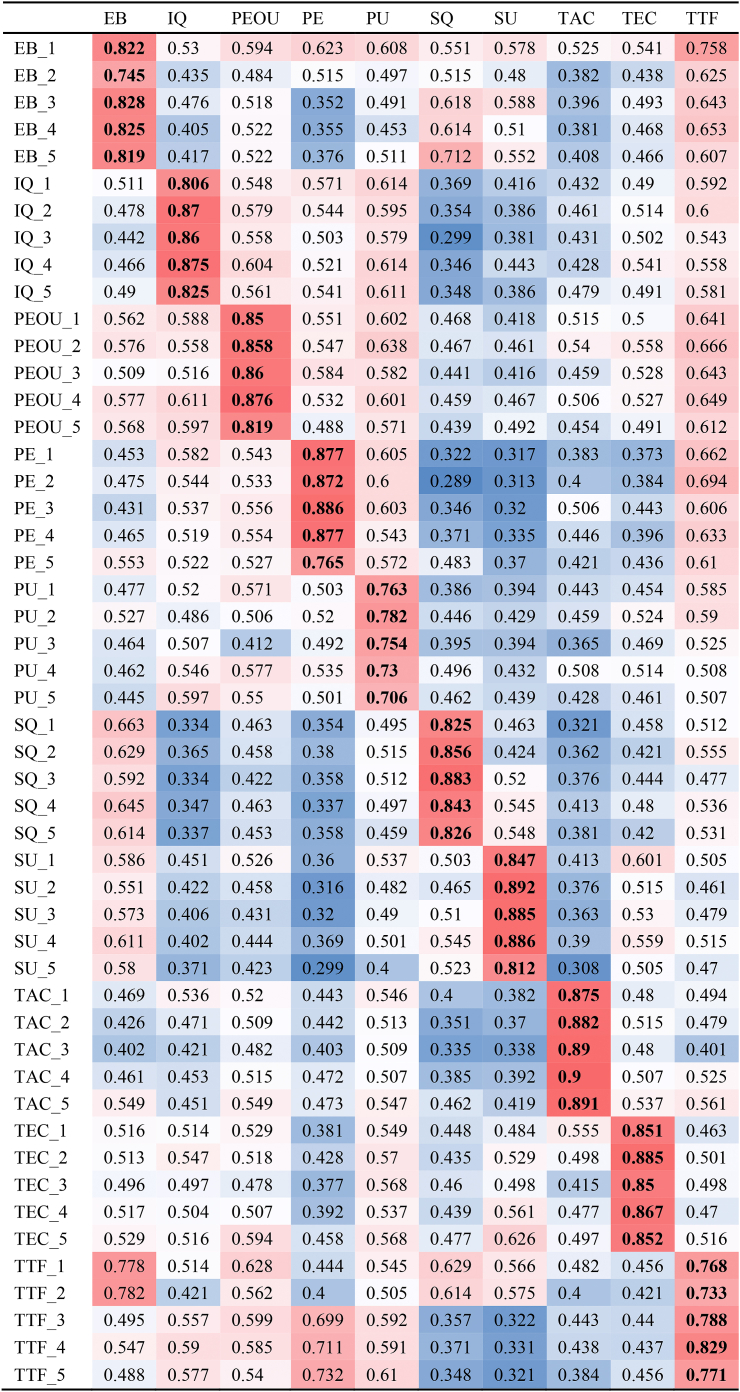
Loading and cross‐loading of measures.

**Table 4:**
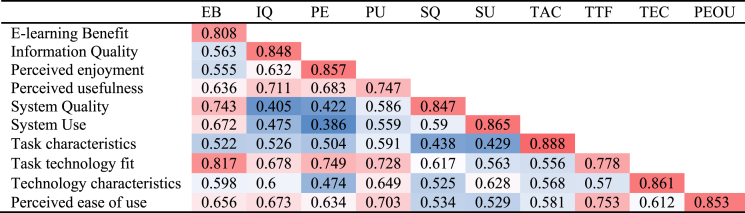
Fornell‐Larcker criterion.

**Table 5:**
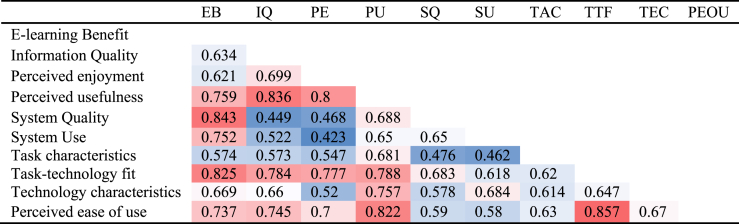
Heterotrait-monotrait ratio for discriminant validity (HTMT).

### 4.4 Measurement model assessment

**Fig. 2:**
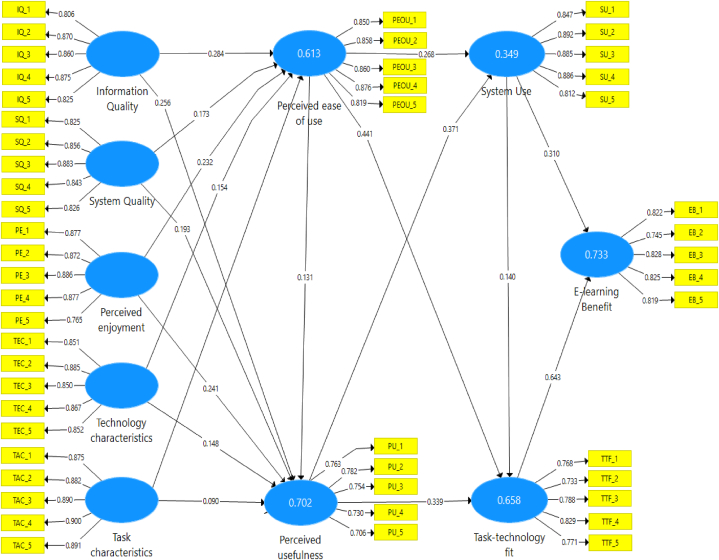
Path coefficient.

**Fig. 3:**
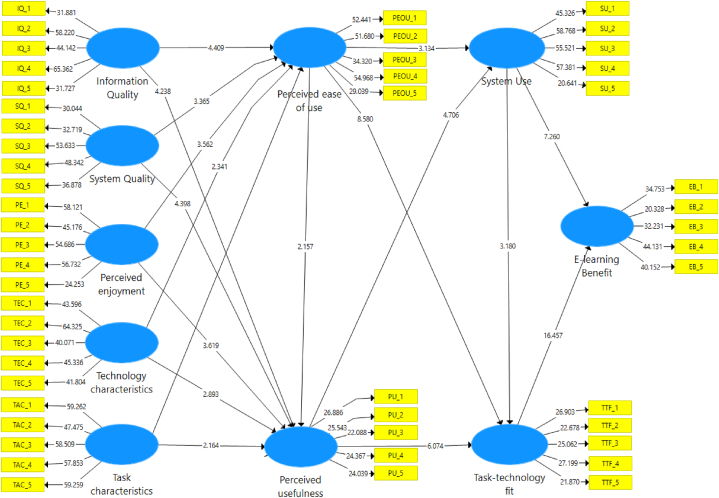
Path (T-Values).

To analyze the structural model, we used a bootstrapping approach with 5000 resamples to assess the path coefficients and coefficient of determination (R2) [110]. Information quality was a positive predictor of perceived usefulness and perceived ease of use, as shown in Table 6. H1 (*P* = 0.284, t = 4.409) and H2 (*P* = 0.256, t = 4.238) were thus supported. Moreover, “system quality” was a positive and significant predictor of PEOU and PU. As a result, H3 (*P* = 0.173, t = 3.365) and H4 (*P* = 0.193, t = 4.398) are supported. Furthermore, perceived enjoyment was a positive and significant predictor of PEOU and PU. H5 (*P* = 0.232, t = 3.562) and H6 (*P* = 0.241, t = 3.619) were thus accepted. Moreover, the significant roles of “technology characteristics” towards perceived use (H7) (*P* = 0.154, t = 2.341) and “technology characteristics” towards perceived usefulness (H8) (*P* = 0.148, t = 2.893) were accepted. In addition, the results for task characteristics and PEOU, which are shown in H9 (*P* = 0.151; t = 2.604), and task characteristics towards PU (H10) (*P* = 0.090; t = 2.164), were accepted. Furthermore, there was support for the relationships between PEOU and PU (H11) (*P* = 0.131, t = 2.157), system use (H12) (*P* = 0.268, t = 3.134), and task-technology fit (H13) (*P* = 0.441, t = 8.580). Furthermore, perceived usefulness (H14) (= 0.441; t = 8.580) and task-technology fit (H15) (*P* = 0.339; t = 6.074) were acceptable predictors of system use. The relationship among system use and task-technology fit, which is H16 (*P* = 0.140; t = 3.180), and system use and e-learning benefit (H17) (*P* = 0.310; t = 7.260), were supported. Finally, the hypothesis that task-technology fit is a significant predictor of e-learning benefit (H18) was supported (*P* = 0.643, t = 16.457). Furthermore, inside the PLS technique, the hypotheses given in Fig. 2, Fig. 3 present the path coefficients and path values (T-value) in Fig. 3 lines. The path values between TTF and the e-learning benefit had the highest t-value (*P* = 0.643; t = 16.457). The correlation between perceived ease of use and perceived usefulness has the lowest value (*P* = 0.131; t = 2.157). In this study, all hypotheses were supported.

**Table 6:** Path, t‐value, and p‐value.

Factors		Path (β)	T- Values	P- Values	Results
Information Quality➔Perceived ease of use	H1	0.284	4.409	0.000	Accepted
Information Quality➔Perceived usefulness	H2	0.256	4.238	0.000	Accepted
System Quality➔Perceived ease of use	H3	0.173	3.365	0.001	Accepted
System Quality➔Perceived usefulness	H4	0.193	4.398	0.000	Accepted
Perceived enjoyment➔Perceived ease of use	H5	0.232	3.562	0.001	Accepted
Perceived enjoyment➔Perceived usefulness	H6	0.241	3.619	0.001	Accepted
Technology characteristics➔Perceived ease of use	H7	0.154	2.341	0.023	Accepted
Technology characteristics➔Perceived usefulness	H8	0.148	2.893	0.006	Accepted
Task characteristics➔Perceived ease of use	H9	0.151	2.604	0.012	Accepted
Task characteristics➔Perceived usefulness	H10	0.090	2.164	0.035	Accepted
Perceived ease of use➔Perceived usefulness	H11	0.131	2.157	0.036	Accepted
Perceived ease of use➔System Use	H12	0.268	3.134	0.003	Accepted
Perceived ease of use➔Task-technology fit	H13	0.441	8.580	0.000	Accepted
Perceived usefulness➔System Use	H14	0.371	4.706	0.000	Accepted
Perceived usefulness➔Task-technology fit	H15	0.339	6.074	0.000	Accepted
System Use➔Task-technology fit	H16	0.140	3.180	0.003	Accepted
System Use➔E-learning Benefit	H17	0.310	7.260	0.000	Accepted
Task-technology fit➔E-learning Benefit	H18	0.643	16.457	0.000	Accepted

## 5 Discussions

Given that TTF and system use both have a positive influence on e-learning benefit, as a result, it can be stated that information quality, system quality, perceived enjoyment, technology characteristics, task characteristics, perceived ease of use, perceived usefulness, task-technology-fit, system quality, and e-learning benefit are all factors to consider. Those with significant effects have a positive influence on e-learning indirectly, through perceived ease of use and perceived usefulness. In fact, individuals have a high level of trust in the e-learning system in terms of information quality, system quality, perceived enjoyment, and technological characteristics. The H1 and H2 hypotheses were accepted, supporting the idea that information quality affects perceived ease of use and perceived usefulness. Since it was discovered that information quality plays a significant role in determining perceived utility and ease of use, e-learning systems should offer more pertinent information to help users achieve their objectives. Our research reveals that e-learners were more engaged in utilizing the e-learning system, which thus raised their reported ease of use and perceived usefulness when they believed the e-learning system could deliver important knowledge for their employment. In order to predict perceived ease of use and perceived usefulness, perceived usability and perceived quality constructs are crucial.

To put it another way, when an e-learning system is impacted by information quality and is appropriate, more usage of the system leads to increased use of the information quality (PEOU) and perceived usefulness for adoption of e-learning systems in higher education. In order to ensure that students have a positive e-learning experience and contribute to their overall satisfaction, it can be said that characteristics of information quality such as providing people with adequate and necessary information, precise and excusable assistance, efficient and up-to-date content, and preferable quality content are important. The study’s findings corroborate those of [117,118], who found that the quality of the information had a substantial impact on PEOU and perceived usefulness. Additionally, the study’s results show that the system quality variable strongly supports the H3 and H4 hypotheses, confirming them with a p-value of (0.001). To put it another way, improved system quality contributes to increased use of perceived ease of use and perceived usefulness for adoption of e-learning systems in education when an e-learning system is simple to use and acceptable (fit). As a result, the ease and usefulness of the typical example are influenced by a number of important characteristics, including the system’s ease of use, ability to meet users' needs, flexibility in engagement, extension and consistency of the different components, and inclusion of capabilities that users need. Our research shows that students perceive an e-learning system’s usefulness in providing helpful functions for efficient learning when it offers high-quality functions to achieve learning goals and tasks and facilitate the learning process. Additionally, if comparable capabilities are offered, students may constantly access course materials, interact with their peers, and communicate with instructors; they will view the system as valuable. These elements influence how easily and effectively students perceive the system to work, which increases their willingness to use it [117,118].

E-learning attracts learners and improves their perceived usability and convenience of use due to the use of video, action, and text type, as well as the speed and capacity of the system. The results of earlier investigations are supported by this study [[119], [120], [121]]. The results of this study for perceived enjoyment H5 and H6 also agree with those of [72,122]. They discovered that perceived enjoyment had a considerable impact on perceived ease of use and perceived usefulness. As a result, both H5 and H6 are supported, demonstrating that perceived enjoyment has a favorable impact on perceived ease of use and perceived usefulness in education. In other respects, increasing perceived enjoyment of an e-learning system results in increased use of the system when it is entertaining and appropriate (fit). The significance of perceived enjoyment in the context of e-learning has been examined in earlier studies. As a result, these study results support past findings [122]. Since using an e-learning system in a way that enhances learning experiences results in satisfaction for learners, this is how perceived enjoyment is defined in the current research.

Previously, hypotheses H7, H8, H9, and H10 were found to have a positive influence on perceived ease of use and perceived usefulness. In the same way, our study confirms that technology characteristics and task characteristics are strongly influenced by students' PEOU and perceived usefulness, which further influences their easefulness and usefulness of e-learning. Alternatively, higher e-learning system use contributes to increased use of the technology characteristics, task characteristics, and perceived ease of use, and PU for adoption of e-learning systems in education. When an e-learning system is impacted by technology characteristics, task characteristics are acceptable. That is, the findings supported all Task-technology-fit model hypotheses. For example, prior research [40,88,123,124] suggested that if an organization wants to develop the acceptance of their employees towards IS/IT, it should target task and technology characteristics and the fit between users' task requirements and information systems/IT functions. Notably, our research discovered that task characteristics had a more significant impact on task-technology-fit than technology characteristics, even though TC has been reported in some studies [40,88] to have a more significant effect on PEOU and PU than TC. Unlike [40,88,105,123], who investigated the effects of specific task activity characteristics (unstructured tasks and teamwork) on task-technology fit, According to the authors, task factors rather than technology characteristics have a significant influence on task-technology fit.

Since students frequently engage in specific task activities during their learning, this study’s findings are consistent with [125] findings that students' learning frequently requires concurrent communication between students and instructors as well as between students. Students also frequently need to easily share, exchange, integrate, and synchronize files and information with other students. This study suggests that the uniqueness of task activities may determine whether task qualities have a bigger direct impact on perceived ease of use and perceived usefulness than technology attributes.

The findings of our study revealed that perceived ease of use is significantly related to perceived usefulness, system use, and task technology fit when e-learning is supposed to be easy, and, hence, users expand their state of mind toward e-learning benefits through task technology fit and system use. To put it another way, when an e-learning system is influenced by PEOU and is acceptable, increased use of the PEOU and PU systems and task-technology suitable for adoption of e-learning systems in learning occurs. Thus supporting H11, H12, H13, H14, and H15. Prior studies supported this conclusion [55,121]. Furthermore, PU was a substantial predictor of system use and task technology fit, and the results were consistent with [126], who discovered that perceived usefulness was strongly associated with attitude in their study. It was found that task-technology fit and the interaction between perceived usefulness and system use were quite important. Students will undoubtedly feel satisfied if they believe that the system and task-technology fit improves their learning effectiveness and operations, enables them to complete their tasks quickly and effortlessly with less effort, and ultimately helps them learn more successfully. Students were more likely to use the e-learning system if they believed it would benefit them.

The results of this study also support earlier research that found PEU has a significant beneficial impact on PU [127], suggesting that e-learning systems with user-friendly features will increase users' PU more than complex systems. The findings also demonstrate that PEU and PU significantly influence system utilization and Task-technology-ft. In contrast, PEOU has a bigger effect on task-technology fit than system use, which has an effect on e-learning. This result is in line with earlier research findings [85,107,108].

Apart from that, some e-learning integration research has found that when respondents believe technology facilitates instructional activities, system use and task-technology fit are more likely to increase [117]. Furthermore, the findings significantly support the system use variable, confirming hypotheses (H16 and H17) and demonstrating that system use positively effects TTF in education as well as the benefits of e-learning. In other words, when an e-learning system is affected by task-technology fit and is acceptable, more system use results in increased use of task-technology fit and e-learning benefits. Students will perform better in the modules, engagement and communication will be simpler, and learning objectives will be accomplished if the use of the e-learning system is in line with the demands of the students. Additionally, the e-learning system will save them time looking for resources and money on things like paper. Previous studies have been done on the significance of e-learning system use. Consequently, this study confirms earlier relationships between variables [55,90,121,[128], [129], [130]].

Furthermore, the outcomes of the study greatly support the task-technology-fit variable, confirming the hypothesis (H18) that TTF in educational institutions and e-learning has benefits. In other words, when an e-learning system is useful and suitable (PEOU and PU), a stronger task-technology fit adds to the increased use of the e-learning benefit and so influences the e-learning benefit. The results show that the benefits of e-learning are primarily dependent on the individual’s own views, familiarity, talents, and self-assurance, more so than on their personality or other people’s views. In addition, task-technology-fit with ISSM criteria such as information quality, system quality, perceived enjoyment, technology characteristics, and task characteristics were found to be important in forecasting technology benefit in the current study. As a result, the study contributes to the current acceptance of information in e-learning by academics in educational institutions, as well as to theory in task-technology fit and information systems success model adoption. The findings can help institutions' administrations, lecturers and teaching staff, and policymakers plan and implement their online strategies, as well as make informed decisions about how to get e-learning recognized by a larger number of local academics. The effective application of e-learning would assist educational institutions in overcoming some of the problems that occur in traditional classrooms. E-learning expands the reach of education beyond time and distance, allowing for better performance monitoring and skill development, which improves output quality and institutional performance. As a result, it is important that educational institution decision-makers evaluate the impact of the above findings when developing plans for increased e-learning benefits.

### 5.1 Theoretical and practical implications

Many researchers have studied the value of TTF in the context of online learning. Therefore, our research supports the existing correlations between variables [55,90,121,129]. By assessing the TTF with ISS Model’s applicability for comprehending the advantages of e-Learning in higher education, these findings add to theory. Furthermore, empirical data were used to support the study framework. The five e-learning benefit dimensions information quality, system quality, perceived enjoyment, technology characteristics, and task characteristics were used as potential constructs that might affect how easy and useful an e-learning system is to use in Saudi Arabia as well as how well it fits into the system as a whole and how well it fits into the task-technology fit. TTF and ISSM models were utilized in this work to measure and assess the results. The following are some of this study’s significant effects and implications:

• It has been established that the ISSM model is the best one for comprehending information quality, system quality in enhancing perceived utility, system use, and task-technology fit. The task-technology fit and system use could ultimately boost the benefits of online learning.
• The TTF model has shown to be a useful tool for understanding students' intentions as they embrace and use e-learning as the medium of e-learning advantage.

Decision-makers in e-learning can benefit from a number of the practical implications of this study. The e-learning system offers decision-makers useful information about the proposed research model and the interrelationships among various decision variables. In order to increase student adoption of e-learning, the current study offers two perspectives. The ISSM and TTF models promote a wide range of priorities to understand student perceptions of ease of use and perceived usefulness, as well as system use and task-technology fit. Test metrics and procedures are also crucial elements in research or practice. Therefore, the model of student acceptance for online learning created in this study can be applied to actual evaluations of e-learning programs created to promote system use. Even though some of the study’s hypotheses aren’t currently supported by any research theories, they are still important to use in quantifying the various aspects of e-learning system usage. Another important theoretical contribution of our research was the successful identification of some data that could be used to fulfil task-technology-fit and system use requirements.

This study offers two lines of evidence: preliminary evidence of perceived usefulness and usability to be employed in the context of felt enjoyment, information quality, system quality, and perceived usefulness. “A technique of perceived ease of use and usefulness that can improve students' acceptance in the context of an e-learning advantage is the second greatest proof of system use and task-technology fit,” according to the study. This is a significant theoretical advance over earlier ISSM and TTF investigations, which did not examine the impact of perceived usability, perceived usefulness, system use, and task-technology fit on the applied ethical aim [85,107,108]. The following are the key deductions made from the study:

• It’s crucial to employ task technology that is appropriate for your learning or to encourage students to accept e-learning systems in higher education. E-learning is one of the key elements of task-technology fit. E-learning should be used more frequently and be more readily available so that all students, no matter where they are, can use it. By correctly responding to student inquiries and simultaneously enhancing the quality of the information through which students can obtain information, the system and information quality may also assist students in their learning.
• Universities typically promote e-learning to students as a means of instruction and learning. In order to streamline the learning process, universities may incorporate all necessary e-learning technologies and content.
• In addition to this, it is indisputably true that employing any form of system raises financial concerns. Universities could therefore take action to ensure that the performance required to enjoy the benefits of e-learning is increased.

## 6 Conclusion

In conclusion, e-learning was widely used as a teaching medium during the COVID-19 epidemic, particularly among students in higher education. The task-technology fit with information systems success model was developed in this study to evaluate the determinants influencing e-learning acceptance at universities during the COVID-19 pandemic. A total of 260 students volunteered to take part in the study and answered a 50-question physical questionnaire. Using partial least squares (PLS), it was found that TTF had the greatest impact on e-learning benefits, followed by PEOU. With these points in mind, all students in higher education should improve their e-learning experience, particularly in terms of educational quality and system quality, while using the eLearning benefit. Variables such as IQ, SQ, PE, TC, and the quality of the eLearning content were also identified as critical indicators that contributed to PEOU and PU. Furthermore, this is the first study to look into the acceptance of eLearning benefits among undergraduate students during the COVID-19 epidemic. These findings could be used by the Commission on Higher Education to develop theoretical guidelines for improving the eLearning system. Although only the task-technology fit with the information systems success model method and one other variable were used in this study, the practical results thus provide strong support for the integrative approach between Tsk-technology fit theory and the ISSM model. The findings suggest an extended TSK-technology fit and ISSM model for the use of e-learning systems to enhance student teaching and learning performance. This model can aid decision-makers in higher education, universities, and colleges in organizing, assessing, and putting into practice the use of e-learning systems. In order to further understand students' views toward the sustainable consumption of e-learning systems.

Task-technology-fit in connection to system use, as determined by the COVID-19, strongly predicts the advantages of e-learning; participants' evaluations of the usefulness of e-learning increase when e-learning is rated as user-friendly. Future scholars who wish to conduct similar studies will require assistance in comprehending the analyses' findings. This study shows that statistical data is available, but it also has significant limitations. Due to the fact that the study’s respondents came from the same institution, future research will need to include additional participants with a variety of majors. However, a few studies have examined the use of e-learning during pandemics like COVID-19. Finally, it is important to recognize and prepare for the study’s limitations. To begin with, there was just one university from which volunteers for this study came. It might affect how broadly applicable the results are. Second, the scope of the analysis was restricted to two possibilities regarding prior expertise in e-learning. On the other hand, there might be extrinsic variables related to system usage and task-technology fit. As a consequence, any future research on the adoption of e-learning should consider other external influences.

## Author contribution statement

Ibrahim AlYoussef: Conceived and designed the experiments; Performed the experiments; Analyzed and interpreted the data; Contributed reagents, materials, analysis tools or data; Wrote the paper.

## Funding statement

This work was supported by the Deanship of Scientific Research, Vice Presidency for Graduate Studies and Scientific Research, King Faisal University, SaudiArabia [Grant No. 2662].

## Data availability statement

Data included in article/supp. material/referenced in article.

## Declaration of interest’s statement

The authors declare no conflict of interest.
