# Supplementary material for: Acceptance of e-learning in higher education: The role of task-technology fit with the information systems success model
Source: Heliyon. 2023 Feb 18;9(3):e13751. doi: 10.1016/j.heliyon.2023.e13751 (PMC9938001; doi:10.1016/j.heliyon.2023.e13751)
Supplement: Multimedia component 1 [file mmc1.docx]

**Questionnaire**

| **Information Quality (IQ)** | | |
| --- | --- | --- |
| 1. | | E-learning provides information that is relevant to my needs. |
| 2. | | E-learning provides comprehensive information. |
| 3. | | E-learning provides information that is exactly what I want. |
| 4. | | E-learning provides me with organized content and information. |
| 5. | | E-learning provides up to date content and information. |
| **System Quality(SQ)** | | |
| 6. | | E-learning provides a proper online assistance and explanation. |
| 7. | | E-learning is aesthetically satisfying. |
| 8. | | E-learning optimizes response time. |
| 9. | | E-learning is user friendly. |
| 10. | | E-learning provides interactive features between users and system |
| **Perceived enjoyment (PE)** | | |
| 11. | I find using the E-learning system to be enjoyable | |
| 12. | The use of the E-learning system stimulates my imagination | |
| 13. | The actual process of using the E-learning is pleasant. | |
| 14. | Using E-learning groups, I have fun sharing knowledge. | |
| 15. | Using E-learning in my study is pleasurable | |
| **Technology characteristics (SEC)** | | |
| 16. | E-learning system can provide me with accessible learning resources anytime and anywhere | |
| 17. | E-learning system can provide me with uninterrupted connectivity and communication anytime and anywhere | |
| 18. | E-learning system can provide me with good folder-sharing and data synchronization function | |
| 19. | E-learning system can allow me to access file /information via internet on different devices and cross operating system platforms | |
| 20. | E-learning system provides useful update status, add photo/video, and post links for education. | |
| **Task characteristics (TC)** | | |
| 21. | My learning often needs to access learning resources anytime and anywhere | |
| 22. | My learning often needs concurrent communication between learners and the instructor and among learners | |
| 23. | My learning often needs file-sharing and information exchanging with other learners | |
| 24. | To my learning, the integration and synchronization of file/  information at different areas and between different devices is highly necessary | |
| 25. | I often need to communicate with other classmates to improve my learning | |
| **Perceived usefulness (PU)** | | |
| 26. | Using the e-learning system improves my course performance. | |
| 27. | Using the e-learning system improves my productivity in courses | |
| 28. | I find the e-learning system useful for my studies | |
| 29. | E-learning helps to save time. | |
| 30. | E-learning helps to save cost | |
| **Perceived ease of use(PEOU)** | | |
| 31 | I find the e-learning system easy to use. | |
| 32. | My interaction with the e-learning system is clear and understandable. | |
| 33. | It would be easy for me to find the required information for using e-learning. | |
| 34. | E-learning is easy to learn. | |
| 35. | E-learning is convenient. | |
| **System use ( SU)** | | |
| 31 | I find the e-learning system easy to use. | |
| 32. | My interaction with the e-learning system is clear and understandable. | |
| 33. | It would be easy for me to find the required information for using e-learning. | |
| 34. | E-learning is easy to learn. | |
| 35. | E-learning is convenient. | |
| **Task-technology fit(TTF)** | | |
| 36 | I think that using e-learning is well suited for the way to learn. | |
| 37. | E-learning is a good tool to provide the way I like to study tasks. | |
| 38. | Using e-learning fits well for the way I like to study tasks. | |
| 39. | I think that using e-learning would be a good way to learn. | |
| 40. | E-learning functions are suitable for helping me complete my coursework. | |
| **E-Learning Benefit (EB)** | | |
| 41 | Using E-learning has increased my knowledge and helped me to be successful. | |
| 42. | E-learning is a very effective educational tool and has helped me to improve my learning process. | |
| 43. | E-learning makes communication easier with the instructor and other classmates. | |
| 44. | E-learning saves my time in searching for materials and cuts down expenditure such as paper cost | |
| 45. | I have control over the E-learning system on campus | |
